# Periprosthetic knee infection after benign tumor excision complicated by carbapenem resistant Enterobacteriaceae: A case report

**DOI:** 10.1016/j.amsu.2022.104292

**Published:** 2022-08-01

**Authors:** Jennifer P. Adams, Daniel Habenicht, Duncan Ramsey

**Affiliations:** a University of Texas Rio Grande Valley School of Medicine, Harlingen, TX, USA; b Department of Orthopedic Surgery, University of Texas Rio Grande Valley School of Medicine, Harlingen, TX, USA

**Keywords:** Periprosthetic infection, Carbapenem resistant enterobacteriaceae, Total knee arthroplasty, Revision arthroplasty

## Abstract

**Introduction:**

Periprosthetic joint infection (PJI) is a major complication after total knee arthroscopy. Enterobacter is a rare cause of PJI.

**Case presentation:**

We present a 65 year old Caucasian man who presented with acute right knee PJI with Carbapenem-resistant Enterobacteriaceae (CRE) two months after undergoing right knee intra-articular mass removal with endoprosthetic reconstruction. The periprosthetic joint infection (PJI) was treated with revision with 1-stage static spacer and IV meropenem.

**Discussion:**

CRE is an uncommon cause of PJI, but when it does occur, it commonly infects patients who are immunosuppressed or have specific risk factors. For an immunocompetent patient with CRE PJI, we suggest further workup for other systemic disease.

**Conclusion:**

This case demonstrates the importance of early diagnosis and treatment of CRE joint infections and the need for a multidisciplinary approach that includes aggressive surgical intervention and tailored antimicrobial therapy.

## Highlights

• We present a case of acute right knee periprosthetic joint infection with Carbapenem-resistant Enterobacteriaceae (CRE) after patient underwent intra-articular mass removal with endoprosthetic reconstruction.
• Further workup for systemic disease is strongly recommended in immunocompetent patients presenting with CRE joint infection.
• Management of CRE joint infections is complicated and requires a multidisciplinary approach with aggressive surgical intervention and focused antimicrobial therapy.

## 1 Introduction

Periprosthetic joint infection (PJI) is a severe complication after joint arthroplasty that is associated with revision surgeries, prolonged hospitalization, and poor outcome [1]. Enterobacteriaceae, ubiquitous gram-negative microorganism found in soil, water, and plants, are part of the intestinal flora in humans. Primary joint infections caused by *E. cloacae*, especially the carbapenem resistant types, are relatively rare but common in the hospital setting in immunocompromised patients and are increasing in incidence due to insurgence of the antibiotic resistance [2].

*E. cloacae* are uncommonly reported as a cause of septic arthritis and the involvement of a prosthetic joint represents a rare event. This is the first case of PJI with *E.cloacae* in an immunocompetent patient without recent bowel instrumentation reported in the literature. We present a case of periprosthetic joint infection (PJI) in an individual who underwent primary right knee arthroscopy after intraarticular tumor removal. Further infectious workup revealed markedly elevated serum C-reactive protein (CRP) and knee culture positive for Carbapenem-resistant *Enterobacter cloacae* (CRE). He underwent prosthesis explantation, debridement, and placement of an antimicrobial mixed spacer.

This work has been reported in line with the SCARE 2020 criteria [9].

## 2 Case Presentation

A 65 year-old patient with a history of longstanding pain, degeneration and slow-growing nodules of the right knee presented to the emergency department on January 2022 with a sudden increase in pain that rendered him unable to function or ambulate. Past surgical history includes esophageal repair in 2009. Family history was noncontributory.

**Fig. 1:**
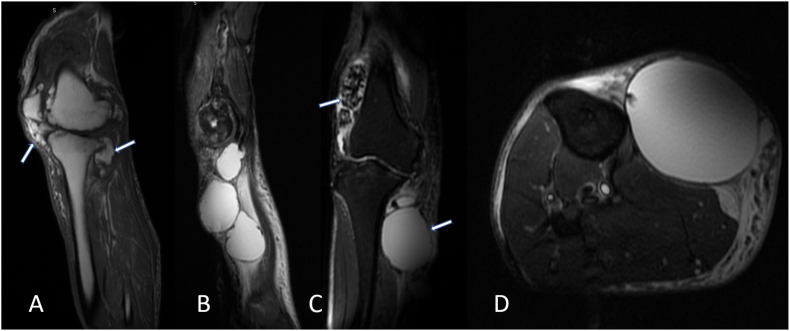
Preoperative MR Images of right lower extremity and knee before total knee arthroplasty. (A) T1 Sagittal view, heterogenous masses communicating with joint space. (B) T2 sagittal view, RLE multiple homogenous cysts. (C) T2 coronal view, heterogenous mass above lateral femoral condyle and cyst abutting proximal tibia. (D) T2 axial view, homogenous cyst abutting proximal tibia.

**Fig. 2:**
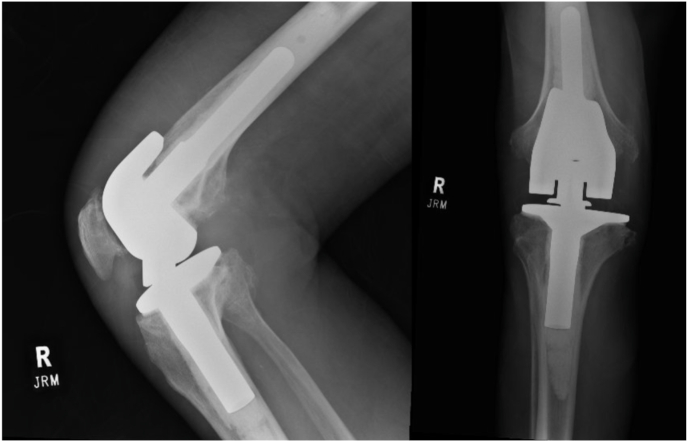
Preoperative Anteroposterior and Lateral radiographic views of knee prosthesis.

Magnetic Resonance (MR) imaging of the right lower extremity (RLE) demonstrated high-grade tricompartmental chondromalacia, excessive synovial osteochondromatosis within the lateral suprapatellar bursa with calcified masses measuring 5.3 cm and 3.7 cm (Fig. 1). Notably, there were three large fluid collections measuring up to 15 cm in the craniocaudal dimension as well as 6 cm in the AP and transverse dimensions. Right knee mass removal, synovectomy, and an uncomplicated reconstruction with endoprosthesis was performed (see Fig. 2).

On March 2022, the patient was readmitted for a 7-day history of worsening burning pain, warmth and swelling of his RLE and right knee. Patient denied fevers, chills, dizziness, chest pain, shortness of breath. On physical exam, two 4cm masses were palpated at the anteromedial leg, RLE nonpitting edema and an area of fluctuance and tenderness to palpation inferior to knee were noted. Preoperative Knee radiography demonstrates loosening of hardware. The right knee was tender and had decreased range of motion. Sensation was intact distally. CRP was elevated at 11.2. Bedside ultrasound of the fluctuant mass revealed loculations and cobblestoning, consistent with infectious etiology. There was concern that this loculated cyst was communicating with joint fluid. Intravenous meropenem was administered and right knee revision with inclusion of a static spacer was performed.

The operation was performed by an orthopedic oncology surgeon and began with an incision into joint space with immediate expulsion of purulent material. Five deep tissue cultures with separate rongeurs were extracted as specimens. Extensive debridement and synovectomy of all diseased tissue was carried out. The distal femur removal step involving freeing the stem proximally was a lengthy process, complicated by an episode of significant bone bleeding. Hemostasis was achieved with sterile tourniquet. For the tibial component, the cement mantle was removed circumferentially and distally. The wound was irrigated with 3 L of bacitracin-infused saline. The spacers were prepared with cement mixed with 3 g of vancomycin and 2.4 of gentamicin and fitted between the femur and tibia. Fluoroscopy was used to confirm proper placement and satisfactory alignment. A 19 Blake drain was placed prior to closure and the arthrotomy was approximated with interrupted sutures. The distal incisions were left open to drain. Intraoperative synovial fluid cultures tested positive for Carbapenem-resistant Enterobacteriaceae (CRE). The patient was continued on meropenem 1 g bolus intravenously over 2 hours, followed by 1 g every 6 hours. The postoperative course was uncomplicated. Over the next month, follow-up in the inpatient rehabilitation unit demonstrated continuous clinical improvements with regressive pain, healed surgical wounds, without signs of persistent infection.

At two months, patient underwent second-stage revision to rotating platform hinge distal femur replacement. At 10-week postoperative exam, patient was without signs of infection. The medical plan was for suppression on levoquin and ambulation pain-free without assistive devices with active ROM 10-100. During admission for reimplantation he was noted by infectious disease specialist to have a subcutaneous 2 cm mass on his upper back which was incised and found to be purulent. He was treated with IV antibiotics for two weeks for this before suppression continued. CRE is thought to be the source of his original infection, as he noted it being there for possibly years, which would explain his atypical species.

## 3 Discussion

PJI occurs in about 2% of primary total knee arthroplasty (TKA) [3]. Approximately 50% of PJIs are caused by *Staphylococcus aureus* or coagulase-negative Staphylococcus epidermidis. Gram-negative bacteria such as the Enterobacteriaceae family are the cause of infection in 3–6% of the cases and are rather uncommon. Microbial contamination of the prosthesis can occur during the operation through airborne contamination or direct inoculation during the handling of the prosthesis intraoperatively [2]. Virulence factors in *Enterobacteriaceae* including adhesins and biofilm formation are major contributors to prosthetic infections. Biofilm is broadly defined as a structured community of microorganisms enclosed in a self-produced matrix, which serve to enhance the bacterial survival inside patients, increasing the probability of causing nosocomial infection [4].

Postoperatively, microbial seeding may occur through hematogenous spread or from contact by an adjacent septic source. One similar reported case was of a 61-year-old immunocompetent man who developed Enterobacter PJI four weeks after a bilateral total knee arthroplasty. The most likely route of the infection was hematogenous seeding of enteric bacteria due to an anastomotic leak after a bariatric surgery done six months before the TKA [5]. For our case, it remains unclear how *E. cloacae* infected the prosthesis of an immunocompetent man who denied history of penetrating trauma or recent abdominal surgery. Imaging before the revision operation revealed severe right knee chondromatosis, reservoirs of excess static joint fluid which, if communicating with surrounding structures, can serve as nidus for infection. It may be beneficial to pursue further diagnostic testing for such patients to identify possible underlying systemic disease or nonobvious viscus leakage permitting entrance of enteric bacteria into bloodstream.

The diagnosis of PJIs can be challenging and is frequently made through indirect measurements of inflammatory markers, joint fluid cell counts and microbiology cultures [6]. The American Academy of Orthopedic Surgeons developed a clinical practice guideline [7] for the assessment of PJI, which include first screening patients who are found to have the potential for PJI with erythrocyte sedimentation rate (ESR) and C-reactive protein (CRP). When one or more inflammatory markers are elevated, an aspiration of the knee to be sent for microbiologic culture and differential white blood cell (WBC) count is then recommended. Prompt diagnosis of knee PJI is crucial to timely surgical intervention. The acute presentation in this report was the main factor influencing the decision to proceed with expedient irrigation and debridement with a one-stage revision.

Treatment algorithms for PJI differ between acute and chronic infections. A two-stage revision is nearly always indicated chronic infections, which involves removal of all hardware, insertion of an antibiotic-loaded dynamic or static spacer, a 6-week course of intravenous (IV) antibiotics, and reimplantation of the joint replacement once the infection is proven eradicated [8] For an acute infection such as the case we described, with symptoms less than three weeks duration, a one-stage revision or irrigation and debridement with retention of hardware is often attempted [8]. There are no established guidelines for the pharmacologic treatment of CRE periprosthetic joint arthritis. Care must be exercised when diagnosing, treating, and preventing CRE as these species of bacteria use diverse moieties to degrade conventional antibiotics. For antibiotic selection, our institution chose meropenem monotherapy with careful monitoring of disease progression and emerging resistance. We suggest that for any periprosthetic infection involving multi-drug resistant species, infectious disease specialist and microbiologist should be consulted as plans should be driven by *in vitro* susceptibility testing.

## 4 Conclusion

To our knowledge, this is the first documented case of Enterobacter *CRE* knee PJI in an immunocompetent patient, with no apparent risk factors such as trauma, diabetes, intravenous substance abuse, malignancy, or immunosuppression. This case highlights the importance of recognizing relevant clinical features and subclinical inflammatory markers to suspect PJI and direct appropriate surgical management. Since Enterobacter PJI occurs more commonly in those who are immunosuppressed, this diagnosis may prompt further workup for other systemic disease. More studies are needed to understand the pathogenesis, role of biofilm, and optimal antibiotic management for carbapenem-resistant microbes in the setting of prosthetic joint infections.

## Patient perspective

N/A.

## Funding

This research did not receive any specific grant from funding agencies in the public, commercial, or not-for-profit sectors.

## Informed consent

Written informed consent was obtained from the patient for publication of this case report and accompanying images. A copy of the written consent is available for review by the Editor-in-Chief of this journal on request.

## Provenance and peer review

Not commissioned, externally peer-reviewed.

## Sources of funding

None.

## Ethical approval

N/A.

## Consent

Obtained.

## Author contribution

JA was involved in the writing of the manuscript. Y and DH were involved in the editing and supervision of the manuscript.

## Research registration (for case reports detailing a new surgical technique or new equipment/technology)

N/A.

## Guarantor

Duncan Ramsey MD.

## Declaration of competing interest

None.
